# Unlocking the potential for achievement of the UN Sustainable Development Goal 2 – ‘Zero Hunger’ – in Africa: targets, strategies, synergies and challenges

**DOI:** 10.29219/fnr.v65.7686

**Published:** 2021-05-26

**Authors:** Prudence Atukunda, Wenche Barth Eide, Kristin R. Kardel, Per Ole Iversen, Ane C. Westerberg

**Affiliations:** 1 Department of Nutrition, University of Oslo, Oslo, Norway; 2 Department of Haematology, Oslo University Hospital, Oslo, Norway; 3 Division of Human Nutrition, Stellenbosch University, Tygerberg, South Africa; 4 Institute of Health Sciences, Kristiania University College, Oslo, Norway; 5 Division of Obstetrics and Gynecology, Oslo University Hospital, Oslo, Norway

**Keywords:** Africa, climate change, Covid pandemic, development programs, food systems, governance, malnutrition, Sustainable Development Goals, United Nations

## Abstract

**Background:**

The UN Sustainable Development Goal (SDG) 2 (‘Zero Hunger’) aims to end all forms of hunger and malnutrition by 2030. Thus, a range of different strategies are needed to facilitate the achievement of SDG 2 to overcome challenges and enable synergies between various SDG targets.

**Objective:**

The aim of this review is to highlight Africa’s progress toward SDG 2, including targets, strategies, synergies and challenges.

**Methods:**

We scrutinized published research articles in peer-reviewed journals, UN reports and in-country Africa reports (between 2015 and 2020) that were relevant to the current topic.

**Results:**

Several hunger indicators are showing slow progress or even deterioration in Africa. The prevalence of undernourishment in the general population was 19.1% in 2019 and is expected to increase to 25.7% by 2030. Improvements in child stunting in several regions in Africa are slow, especially in sub-Saharan Africa where about 34% of under-fives were stunted in 2012 and 31% in 2019. In Eastern Africa, stunting prevalence decreased from 38% in 2012 to 34% in 2019. Major drivers of hunger are poor governance and state fragility, war and conflicts, increasing inequality, weak economic development, climate change, biodegradation – and now lately the Covid 19 pandemic – factors that all increase food insecurity.

**Conclusion:**

Africa is off track to reach SDG – ‘Zero Hunger’ – by 2030. Current efforts and progress are insufficient. Africa must champion the SDG agenda on a national, regional and global level to facilitate synergies to unlock the potential for reaching ‘Zero Hunger’ throughout the continent.

## Popular scientific summary

- The UN Sustainable Development Goal (SDG) 2 (‘Zero Hunger’) aims to end all forms of hunger by 2030.
- Africa is off track to reach this goal. Current efforts and progress are insufficient.
- Major drivers of hunger are war/conflicts, poor governance, inadequate health services, increasing inequality, weak economic development, climate change and biodegradation.
- Africa must champion the SDG agenda on all levels and facilitate synergies to unlock the potential for reaching ‘Zero Hunger’ throughout the continent.

## The UN Sustainable Development Goals

**Fig. 1:**
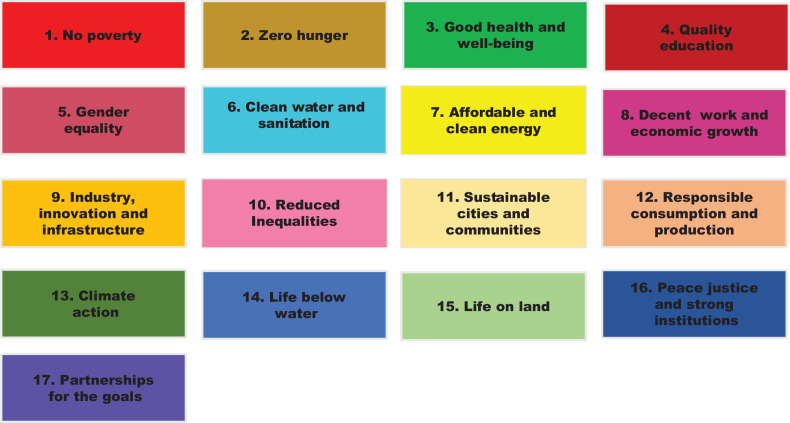
An overview of the 17 United Nations Sustainable Development Goals. Modified from (65, 66).

The Sustainable Development Goals (SDGs) were born at the United Nations (UN) Conference on Sustainable Development in Rio de Janeiro in 2012 where the main objective was to produce a set of universal goals that met the urgent environmental, political, and economic challenges facing the world (1). Notably, 17 SDGs were to replace the eight UN Millennium Development Goals (MDGs) (Fig. 1). This marked the start of a renewed and collective global effort to tackle the indignity of poverty, especially in low- and middle-income countries (LMICs), as well as an acknowledgment of the need for the human species to adjust its living patterns within sustainable planetary boundaries. Thus, the SDGs represented a considerable advance from the MDGs, with a substantially broader agenda affecting all nations, requiring coordinated and sustainable global actions (2). The processes toward the 17 SDGs were led by the nations rather than steered by international agencies as was the case with the MDGs. The UN Member States themselves guided the whole SDG process, including leading discussions and the selection of goals, targets, and indicators (3).

**Fig. 2:**
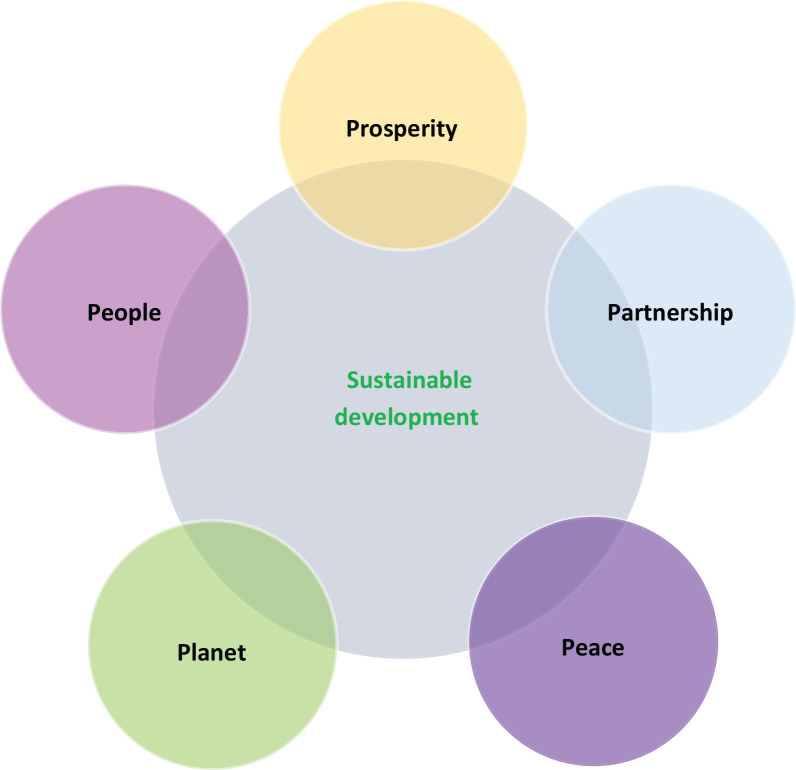
The 5Ps (people’s well-being; planet with protection of the earth’s ecosystems; prosperity with eradication of poverty and inequality; peace, and international partnerships) of sustainable development. Modified from (4, 5).

Within the wide thematic areas covered, the SDGs’ core focus is on the cross-cutting 5 Ps (Fig. 2): People’s well-being; Planet with protection of the earth’s ecosystems; Prosperity with eradication of poverty and inequality; Peace, and international Partnerships (4, 5).

### SDG 2 ‘Zero Hunger’ – targets and indicators

SDG 2 ‘Zero Hunger’ includes several targets to be achieved by 2030. To monitor the progress of these targets, several indicators are defined (Table 1). The targets and indicators for SDG2 ‘Zero Hunger’ include nutrition, food production, agriculture, and food systems (6).

**Table 1:** Sustainable Development Goal 2 (‘Zero Hunger’) – targets and indicators relevant for Africa

Sustainable Development Goal 2: Targets	Sustainable Development Goal 2: Indicators
Target 2.1: Universal access to safe and nutritious food	2.1.1 Prevalence of undernourishment 2.1.2 Prevalence of moderate or severe food insecurity in the population, based on the Food Insecurity Experience Scale
Target 2.2: End all forms of malnutrition	2.2.1 Prevalence of stunting among children under 5 years of age 2.2.2 Prevalence of childhood malnutrition (wasting or overweight)
Target 2.3: Double the productivity and incomes of small-scale food producers	2.3.1 Volume of production per labor unit by classes of farming/pastoral/forestry enterprise size 2.3.2 Average income of small-scale food producers, by sex and indigenous status
Target 2.4: Sustainable food production and resilient agricultural practices	2.4.1 Proportion of agricultural area under productive and sustainable agriculture
Target 2.5: Maintain the genetic diversity in food production	2.5.1 Number of plant and animal genetic resources for food and agriculture secured in either medium- or long-term conservation facilities 2.5.2 Proportion of local breeds classified as being at risk, not at risk, or at unknown level of risk of extinction Target for these two indicators is set for the year 2020
Target 2.A: Invest in rural infrastructure, agricultural research, technology, and gene banks	2.A.1 Agriculture orientation index for government expenditures 2.A.2 Total official flows (official development assistance plus other official flows) to the agriculture sector
Target 2.B: Prevent agricultural trade restrictions, market distortions, and export subsidies	2.B.1 Value of agricultural export subsidies
Target 2.C: Ensure stable food commodity markets and timely access to information	2.C.1 Indicator of food price anomalies

### The current hunger situation in Africa

**Fig 3:**
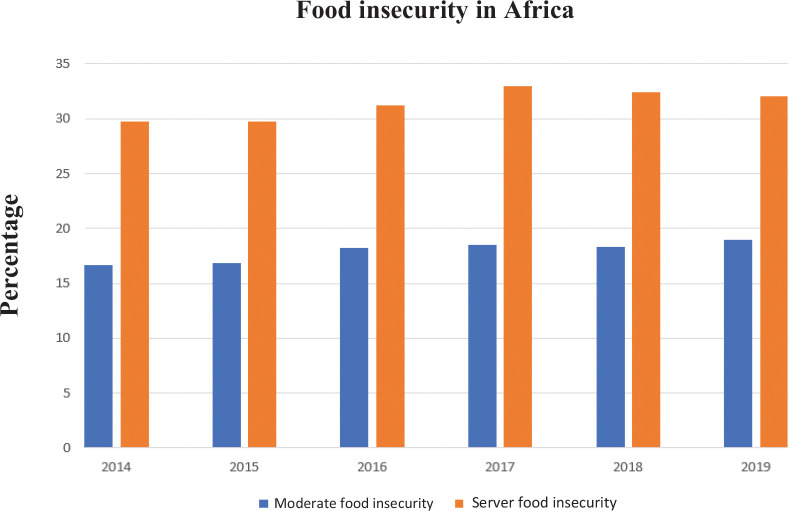
Food insecurity in Africa for the period 2014–2019. Modified from (8).

The prevalence of *undernourishment* is a ‘Zero Hunger’ indicator using in-country data to estimate dietary energy intakes relative to food availability based on national food balance sheets. Since 2015, the prevalence of undernourishment, which is defined as an estimate of the percentage of the population whose habitual food consumption is insufficient to provide the dietary energy levels required to maintain a normal active and healthy life, is gradually increasing (7). The majority of undernourished people in Africa is found in the sub-Saharan region, which shows an increase of about 32 million undernourished people since 2015 (8). Table 2 shows the prevalence of general population undernourishment in African regions for 2005–2019, with only Northern Africa showing a decline. It is discouraging for Africa that a rapid increase in the prevalence of undernourishment is projected from 19.1% in 2019 to 25.7% in 2030 (8). Notably, when measured using the food insecurity experience scale (FIES), the state of both moderate and severe food insecurity increased in Africa as a whole from 2014 to 2019 (8) (Fig. 3). FIES is a self-reported questionnaire focusing on behaviors and experiences related to difficulties accessing food because of resource constraints.

**Table 2:** Prevalence of population undernourishment in the world and in African regions

Prevalence of general population undernourishment (%)^a^								
	2005	2010	2015	2016	2017	2018	2019^b^	2030^b^
World	12.6	9.6	8.9	8.8	8.7	8.9	8.9	9.8
Africa	21.0	18.9	18.3	18.5	18.6	18.6	19.1	25.7
Sub-Saharan Africa	23.9	21.3	21.2	21.4	21.4	21.4	22.0	29.4
Eastern Africa	32.2	28.9	26.9	27.1	26.8	26.7	27.2	33.6
Middle Africa	35.5	30.4	28.2	28.8	28.7	29.0	29.8	38.0
Southern Africa	4.9	5.4	7.0	8.0	7.0	7.9	8.4	14.6
Western Africa	13.8	12.1	14.3	14.2	14.6	14.3	15.2	23.0
Northern Africa	9.8	8.8	6.2	6.3	6.6	6.3	6.5	7.4

a The prevalence is defined as an estimate of the proportion of the population whose habitual food consumption is insufficient to provide the dietary energy levels required to maintain a normal active and healthy life. Modified from (7).

b These are projected values.

*Stunting* among children below 5 years is commonly used as a marker of chronic undernutrition and defined as height-for-age z-score more than two standard deviations (SD) below the World Health Organization (WHO) Child Growth Standards median. Stunting is a result of insufficient nutrient and energy intake over a long period of time. There are large variations of stunting prevalence both between and within countries. In 2019, 144 million children under 5 years were affected by stunting worldwide, a 12% reduction relative to the baseline reference year for the global nutrition targets in 2012 (164 million) (9, 10). In Africa, from 2000 to 2020, there has been a gradual decline in the number of stunted children below 5 years of age, from 38 to 29% (9, 10). However, the prevalence is still high and even short of the set targets for both 2025 (about 16%) and may still not be achieved by 2030 set target of 11% (8). The biggest challenge remains in sub-Saharan Africa which is the only region with increasing prevalence of stunting (8). In line with this, the highest stunting prevalence in 2014 was found in East Africa (43%) according to the UN (9), whereas a recent analysis reported a stunting prevalence of 33% among under-fives children under 5 years (11). Notably, there were wide variations between countries in East Africa, ranging from 21.9% in Kenya to 53% in Burundi.

*Wasting* (weight-for-height z-score < –2 SD of the WHO Child Growth Standards median) among children under 5 years, which is a marker of acute malnutrition, is still way above the set global targets, that is, 6.4% in 2019, 5% in 2025 and 3% by 2030 (8). Currently, the prevalence of wasting for the African region is 6.4%, with only the Southern African region having a prevalence below 5% (9, 10).

### Strategies to combat hunger toward SDG 2 – a mixed African perspective

As to African strategies to achieve ‘Zero Hunger’, there is no one-type-fits–all approach. The continent offers widely different conditions for land-, water- and forest-based food production with regard to soil and rainfall. The level of technological development, besides economic, legal, and social assets of cultivators, varies among fisher folks, forest people, and nomadic groups to achieve food security.

#### Peace, governance, and institutions

The 2019 Global Report on Food Crisis indicated that conflict created food insecurity in the following African countries: Democratic Republic of Congo, South Sudan, the Lake Chad Basin, Somali, and the Central African Republic (12, 13). Therefore, African countries must establish political and financial commitment to proposed SDGs actions backed by institutional reforms, strict implementation measures, as well as quality monitoring and evaluation of progress (14). In 2015, UN food agencies suggested that poverty and hunger eradication in all LMICs by 2030 would be possible (15). This would involve investments in social protection combined with public and private efforts to raise investment levels in productive sectors, especially among rural areas and particularly in agriculture (16).

#### Food systems and agriculture

Robust food systems and agriculture are crucial for food security. Agricultural diversification means growing individual food crops for consumption and where possible keeping a variety of animals for meat or milk and eggs (17). Climate Smart Agriculture is a strategy that includes development and promotion of innovations to adapt and create resilience to climate change and extreme weather events (18). This focuses on the use of high-yielding, drought-tolerant crop varieties, climate information services, agricultural insurance, agroforestry, water harvesting techniques, and integrated soil fertility management practices. Such climate smart agriculture will be especially important in West, Central East, and Southern African regions that are prone to challenging climatic conditions.

Africa must ensure that food production strategies are based on solid policy frameworks that safeguard food security to rural communities as well as sustainable production. Two dominant types of agricultural production systems stand in contrast to each other and may cause political tension. Simplified, the commercially based large-scale mono-crop intensified cultivation is geared to increase yields aimed primarily for the foreign markets, typically generated by multinational corporations. These often buy land and squeeze smallholder farmers out of production. On the other hand, various forms of locally based and diversified food production by small farmers have proven to be successful in both enabling healthy diets and often some income for the households, but may lose in the competition with large food-chain companies due to lack of appropriate markets. Over the last decade, the understanding of the potential of smallholder farming also with respect to yields comparable to high-tech commercial agriculture has increased. Agro-ecological farming, drawing on local environmental condition, traditionally successful farming methods, safeguarding of ecosystems and capturing carbon, has attracted renewed interest. Less use of artificial fertilizers and pesticides brings the best of traditional knowledge to the fore as well as new research aimed at improving food security to rural families (19).

#### Collaboration with UN specialized agencies, programs, and funds

African countries are collaborating with several UN development bodies to achieve ‘Zero Hunger’, including various activities explicitly aimed at ending hunger and achieving sustainable food systems in Africa (20). Some token examples are listed in Table 3.

**Table 3:** Examples of UN-based initiatives directed towards achieving Sustainable Development Goal 2 in Africa

UN Agency	‘Zero Hunger’ initiatives	Implemented case examples	Location in Africa
United Nations Development Programme (UNDP)	Women first initiatives focusing on women emancipation, livelihood diversification, for example, changes in social norms to avoid excluding women from resource ownership and property (57)	Collective gardens, for example Rain4Sahara where women are growing food for cash, hygiene, and nutrition programs From subsistence to sustenance, women provided with extension agents and farming services to grow nutritious diverse fruits and vegetables	Niger, Sahel region Ethiopia, Horn of Africa
Food and Agriculture Organization (FAO)	Skilling of smallholder rural farmers Farmer notebook focusing on conservation agriculture (58) Funding of SDG 2, promotion policies (91% of FAO project portfolio and budget of about 6.7 million USD) (59)	Solar dried vegetables Education on good farming practices to reduce soil erosion, moisture loss, and conserve soil nutrients Supports national programs, legal and policy initiatives, regional programs, investment in food systems, climate finance, UN collaboration, and education. Farmer field schools, support communities in the implementation of good agricultural practices to boost incomes	Kenya, East Africa Zimbabwe, South Africa Across Africa Burkina Faso, Nigeria, West Africa. Kenya, Malawi, Uganda, East Africa
World Food Programme (WFP)	Humanitarian food assistance Scaling-up nutrition investments in food security, procurement, and logistics Educating communities about the nutritional importance of eating a wide range of foods (60)	Put the most vulnerable first Pave the road from farm to market Encourage sustainable variety of crops	All regions of Africa All regions of Africa All regions of Africa
International Fund for Agricultural Development (IFAD)	Nutrition-sensitive agriculture Investing in rural agriculture (61) Rural women finance projects (61)	Agribusiness/value-chain development Rural marketing	Zambia, Southern Africa Mozambique, Southern Africa Zambia, Burkina Faso, Ghana, Mali, Gambia, Benin and Sierra Leone
UN Committee on World Food Security and Nutrition- High Level Panel of Experts (HLPE)	Global evidence-based international and intergovernmental scientific platform Facilitate policy debates and policymaking (62)	Expert reports on different aspects of food security and nutrition Providing independent, comprehensive analysis on effective policy frameworks for fundamental food security systems transformation	All regions in Africa
The United Nations Children’s Fund (UNICEF) and World Health Organization (WHO)	Food systems approaches that affect food, people, and the planet (63) Developing guidelines on healthy, nutritious, and sustainable diets to promote child full potential development (64)	Framework on Food Systems for Children and Adolescents UNICEF continues to support nutrition programs focusing on prevention first and, if that fails, then treatment	All regions in Africa All regions in Africa

Also to be mentioned in this context is the recent (February 2021) adoption of the ‘UN Voluntary Guidelines on Food Systems and Nutrition’ by the UN Committee on World Food Security. Here, experiences from and recommendations for ways to reach SDG 2 at an accelerated pace toward 2030 were negotiated. Alongside this more than 2 years long process, an initiative of the UN Secretary-General in 2019 for a UN Food Systems Summit in September 2021 is being planned in various ways, including through five separate ‘Action Tracks’ directed by experts in various fields together with interested states, civil society and private sector organizations, and with opportunities for wide engagement for those interested in proposing various ‘game-changing solutions’ toward achieving SDG2. Also, national dialogues in some 60 countries, both government-led and independent ones, will provide new information of the efforts of individual African states regarding what ought to be more coordinated efforts at national levels toward 2030.

The UN has been integrated into nationally led SDG implementation strategies to end hunger through the Zero Hunger Challenge. Each UN development agency is aligned with the 2030 Agenda in their programs and activities to deliver outcomes across a range of the agenda’s goals and targets to attain ‘Zero Hunger’ in Africa. They are committed to bring together governments, civil society, the private sector, and others for collective impact in the area of food security, nutrition, and sustainable food systems (21). This collective commitment focuses on a call to end hunger, eliminate all forms of malnutrition, and build inclusive and sustainable food systems within planetary safe operating limits.

## Synergies among the SDGs to achieve ‘Zero Hunger’

An important recognition underlying the SDGs is that all the 17 goals are interrelated, in what is commonly known as a synergistic relationship. Thus, success in one goal affects the attainment of others, including SDG 2 ‘Zero Hunger’. For example, SDG 1 ‘No Poverty’ has impact on the ‘Zero Hunger’ target since being poor negatively impacts the capacity of individuals to access adequate food both in quality and in quantity (22). Synergy between SDG 3 ‘Good Health and Well-Being’ and SDG 2 is through the fact that good health is closely linked to nutrition, with good health relying on sufficient and adequate macro- and micronutrient intake. Furthermore, access to quality health care is necessary to prevent and treat diseases that may increase nutrient needs through increased catabolism and malabsorption. Furthermore, SDG 3 ‘Good Health and Well-being’ includes family planning and reproductive health that is important to ensure manageable family size, which is less prone to food insecurity and improved child nutritional status (23). SDG 4 on ‘Quality Education’ relies on the achievement of ‘Zero Hunger’ since food is needed to facilitate learning and cognitive development. In the long-term perspective, quality education and learning enable individuals and societies to develop. In many countries, school meals are an important source of daily nutrition and good health promotion (24). In fact, a quality education should include a curriculum involving food, nutrition, and agricultural food production, targets within SDG 2. SDG 5 ‘Gender Equality’ is important to achieve ‘Zero Hunger’ since women with access to income typically improve the children’s nutrition and health (22). SDG 6 ‘Clean Water and Sanitation’ is a prerequisite for good health through avoidance of enteric infections. In fact, not having access to safe water impacts other parts of daily life activities, including nutrition. For example, lack of clean water for drinking and to prepare food increases the vulnerability to infections and sickness, which in turn affects nutritional status (25). SDG 8 ‘Decent Work and Economic Growth’ is related to the ‘Zero Hunger’ target since countries with high rates of malnutrition and food insecurity may have a high loss of gross domestic product (26). SDG 13 ‘Climate Action’ is of major importance, especially to some of Africa’s most vulnerable societies, since climate change increases droughts, floods, and other extreme weather events, with detrimental effects on food production (27). SDG 14 ‘Life Below Water’ and SDG 15 ‘Life on Land’ relate to loss of biodiversity, acidification of the oceans, and soil degradation, which threaten the ability to produce food (22). SDG 16 ‘Peace, Justice and Strong Institutions’ is critical to ensure ‘Zero Hunger’ as instability, war, and bad governance are among the major contributors of food and nutritional insecurity. SDG 17 ‘Partnerships for the Goals’ pinpoints the necessity for strong global collaboration to achieve the goals, including SDG 2 ‘Zero Hunger’ (22).

### Challenges to achieve SDG 2 in Africa

Several challenges are slowing down the progress to achieve the desired targets, which are discussed next.

#### State fragility

Conflicts, wars, and insurgencies have affected many African countries, resulting in several conflict events at one time or another, rendering many hungry and food insecure. For example, in 2017, the South-Sudan war caused 42% of its population to face severe food insecurity (28, 29). Somalia, South Sudan, Chad, and the Democratic Republic of Congo, known for protracted crises, have very high child undernourishment and under-five mortality rates compared to stable nations in Africa (29).

#### Poor governance and corruption

In Africa, poor governance by far has hampered the progress of food security in many nations. Governance is a key priority action area to mitigate food insecurity in terms of building and enabling policies and regulatory frameworks to enhance increased coordination of agricultural, climate change, and food system policies (30). Due to poor governance, policy, and coordination of national agricultural policies, strategies, investment plans, and climate change instruments, including national adaptation programs, are lacking in many food insecure African countries (30).

Poor governance and corruption, specifically the lack of democracy in food and agriculture, widen the already yawning gap between the have and have-nots in many African countries. Thus, no initiative on food security will work in the absence of ethical public behavior because of corruption and poor governance culture (31). Increased reported corruption in the import and distribution of agricultural inputs by government agencies in African countries delays the end to hunger goals (31).

#### Climate change

Climate shocks, as evidenced by the increasingly more frequent occurrences of cyclones and droughts, have affected the most vulnerable populations in Africa through devastating effects on their food and nutrition security. The estimated numbers of droughts and floods have increased, respectively, from 89,256,000 and 5,583,000 between 1980 and 1989 to 158,509,000 and 23,332,000 between 2000 and 2009 (32). Regrettably, greenhouse gas emissions, attributed mostly to the industrialized Western World, are linked to adverse climate changes, causing food insecurity for the poorest people in the global south hardest, mostly in LMICs (16). Water shortages are the most concerning aspect of climate change in Africa. Already, in parts of the Sahel region such as Mali, desertification is reducing available croplands (8). Furthermore, climate change affects local biodiversity and may contribute to new invading alien species affecting local food production. This is currently a large problem with a locust invasion in Eastern Africa that threatens to eradicate crucial harvests from the local small-scale farmers (33). A predictive model on climate change including possible determinants projects a 20% increase in child malnutrition by 2050, and a 50% decrease in crop yields in many sub-Saharan countries (32). Unfortunately, this model mentions that by 2080s, arid and semi-arid land in Africa will have increased by 5–8%, leading to significant reduction in rain-fed land for cereal production. Thus, the success of the SDG 13 ‘Climate Action’ and the Paris Climate Accord (target of staying below 2°C warming) and the future temperature trajectory will be of major importance to food security on the African continent. Furthermore, increased competition for key resources, such as fertile land and clean water, contributes to provoking violence and armed conflicts, exacerbating the vicious circle of hunger and poverty and resulting in protracted crises.

#### Natural resource mismanagement

Mismanagement of natural resources, such as water, largely contributes to food insecurity and inefficacy of food production practices. African countries that have prioritized good practices and technologies utilizing efficiency in water use and management have promoted their food productivity gains, as evidenced by outcomes of research and development (R&D) investments. African countries that have made significant investments in agricultural R&D continue to reap food productivity and security gains for their population. For example, Namibia, largely a desert country, utilizes the available water resources to enhance food production by having a system of responsive and accountable governance (34).

#### The role of forests for food security, nutrition, and the challenge of forest mismanagement

In 2017, the High Level Panel of Experts of the UN Committee on World Food Security emphasised that sustainable forest management is important to maintain and enhance the economic, social, and environmental values of all types of forests (35). This is important in the strive toward ‘Zero Hunger’ since deforestation is a critical sustainable development challenge, as increasing food production to meet growing demand has strikingly reduced tropical forests. This is especially true in sub-Saharan Africa that continues to face serious food insecurity issues because smallholder farming is the main driver of forest reduction (36). During the period between 2001 and 2015, 92% loss of land area covered by forests in Africa was attributable to expansion of smallholder farming (37). For example, the Democratic Republic of Congo and Cameroon have reported increasing deforestation associated with high levels of poverty and food insecurity (38). Annually, about 13 million hectares of forests are lost due to deforestation, partly by agriculture, logging, mining, and infrastructure development. Deforestation is a significant factor in promoting climate change through increased emission of greenhouse gases, thus altering temperature and weather patterns globally (27, 39).

#### Fisheries and aquaculture

Fisheries and aquaculture have often been arbitrarily separated from other parts of the food and agricultural systems in food security studies, debates, and policymaking. Small-scale fisheries in sub-Saharan Africa are threatened by overfishing, pollution, and competition for water which is a potential threat to their sustainability. The significant development of aquaculture raises many questions about its environmental impacts on land, water, and biodiversity, as well as sustainability, and has itself to face competition from other users of land (40). However, the demand for fish is growing due to a combination of factors such as population growth, urbanization, and increasing wealth and incomes. Aquaculture is one of the few food production sectors worldwide where growth in production is outpacing growth in population. Small-scale fisheries can give opportunities to the poorest, landless, food-insecure people and households, providing them a critical (and sometimes unique) source of income and livelihood. Intake of fish can help reduce the risks of malnutrition and of non-communicable diseases. Farmed fish contribute to improved nutritional status of households, directly through self-consumption, and indirectly through selling farmed fish to enhance household purchasing power. Notably, there is almost a consensus that women’s roles in aquaculture and fisheries are not fully recognized, often go unrecorded, are undervalued, and are largely invisible in national statistics (41). Thus, given the importance of small-scale fisheries and aquaculture in poverty alleviation, food security, and nutrition in sub-Saharan Africa, governments should make fish an integral component of inter-sectoral national food security and nutrition programs, with special emphasis on small-scale capture fisheries and fish farming or aquaculture projects. Stakeholders in these fish subsectors should support self-organized local professional organizations and cooperatives, as these strongly contribute to and foster the integration of small-scale operations into markets. State labour, finance, and policy formulation and implementation agencies, in collaboration with fisheries agencies, should improve national regulations for fish workers, including women workers in fish processing factories and markets, ensure that adequate and specific budget allocations are made for small-scale fisheries and aquaculture development, and facilitate the direct involvement of farmers and other stakeholders in the process of priority setting and choice of technology (41).

#### A food system for the first 6 months of life and beyond

As recommended by the WHO, infants should be given exclusively breastmilk for the first 6 months of life and if possible continue with breastfeeding for up to 2 years or beyond in combination with suitable complementary foods. The interactive food systems implied have numerous dimensions economically, socially, culturally and psychologically in relation to women’s lives and rights. While breastfeeding mothers are the primary actors to ensure an ideal and functioning food system in this case, they need support at many levels: near family, practice at delivery wards, community support, workplace allowances, legal protection, etc., to maintain the protective food system needed through these critical first 1,000 days of life. Any government must decide whether it will actively promote and strengthen this critical food system with regard to actively protecting and optimizing nutrient supply to its youngest citizens. The constant and often misleading advertisements of breastmilk substitutes for profit by large multinational companies must be met by strong legal regulations of such marketing and through systematic follow-up. Moreover, the International Code of Marketing of Breast-milk Substitutes and subsequent biannual resolutions should be implemented in national legislations (42). The stakes are high, as ‘Zero Hunger’ not least demands action for the particularly vulnerable age bracket of 0–24 months. The issue should therefore no longer be dealt with by health systems and especially primary health care alone as is typical, but be treated alongside other food system challenges in their own right.

#### Conflicting global and national food policies

Global and national food policies affect food security in both rural and urban Africa. Despite food insecurity generally having been described as a rural issue, the effect of global and national policies on food insecurity is increasingly making it an urban challenge (43). Increased export-driven agriculture by big companies may cause small-scale farmers to sell their farm land and migrate to cities. Thus, the functioning of the food systems is an increasingly central issue for policymakers concerned with the future development of urban areas in Africa (44). ‘Super marketization’ refers to the fact that big supermarkets have taken over retail shops in many African countries. There are several concerns over the negative impacts of the ‘super marketization’ of food. They may provide cheaper food, and their contribution to dietary shifts escalates the ‘triple burden of malnutrition’ (the coexistence of overweight/obesity, undernutrition, and micronutrient deficiencies) (45). ‘The global supermarket’ is another term reflecting the dominant power of transnational food companies that often control whole food chains from production to retail level. Combined with frequent unethical marketing of ultra-processed unhealthy foods to children and youth, these companies play a role in the global ‘nutrition transition’ from traditional foods to mote-processed, energy-dense, nutrient-poor foods that contribute to increasing overweight/obesity also in many African countries.

#### Conflicting agricultural practices

Conflicting agricultural practices and policies have affected progress toward ‘Zero Hunger’ in Africa. The choice between organic and conventional agriculture may impact health. For example, biofertilizers in conventional farming containing heavy metals have shown an extremely long persistence in the soil environment, leading to metal accumulation (46). In addition, mechanized farms in sub-Saharan Africa may increase gender inequalities especially among rural women in some countries (47). This can be observed in terms of loss of employment for casual laborers, when low-income rural workers are being replaced by mechanization, which in turn threatens their food security and nutritional status. In Zimbabwe and Malawi, reports indicated negative effects of inadequate conservation agriculture (improved soil structure and soil erosion protection) to promote food security among farmers (48). Notably, the majority of rural smallholders are less likely to benefit from mechanization of agriculture. For example, small-scale traditional farmers cannot afford tractors even when they are highly subsidized, which leads to elite capture (31). A case example is Ghana, were it was found that distribution of government-imported tractors was not transparent and encouraged rent-seeking behavior (31). Sadly, the tractor imports were politically more attractive than investing in skills development (49). Tractors showed short-term effects and generated media attention, which was particularly valuable prior to elections just as in many other African countries (31).

Good agricultural practices, including the importance of food security initiatives in school curriculum, are desired, for example, through *in situ* soil application and utilization of organic waste materials via compost processing (50). This has been shown to enhance soil and plant productivity, increase soil water retention, sequester carbon, and decrease external synthetic fertilizer and chemical inputs. Such an educational model for organic waste-to-resource initiatives is positively associated with food production for long-term sustainability that is in alignment with SDG 2 ‘Zero Hunger’ (50).

Another conflicting food policy in Africa is the imbalance between agricultural extension/agricultural advisory services in the interest of large commercial producers versus those of family farmers. For example, a recent Ethiopian study identified gaps between digitalizing agricultural extension information services and stakeholders’ experiences (51).

#### Population growth

Population growth will greatly increase the amount of food needed to adequately feed Africa’s people. Despite a fall in fertility rates, the number of children per family in Africa is still much higher than the global average (2.4 children per woman in 2018), and the population is growing. For example, in sub-Saharan countries, the fertility rate has gone from 6.8 children per woman in the 1970s to 4.7 children in 2018 (52). Increasing population pressure in Africa (about 1.2 billion in 2018) has impacts on food security and has worsened land scarcity, land use intensification, and land degradation linked to food insecurity. This together with increased poverty is associated with a stronger tendency to use soil-mining practices, for example, removal of soil conservation structures to use fertile soils within the structures (53). Remarkably, land degradation in combination with population growth continuously leads to increasing food insecurity unless targeted policy interventions for improved food markets and agro-processing technology adoptions are introduced (53).

#### The coronavirus disease 2019 (COVID-19) pandemic

The COVID-19 pandemic is here singled out as a challenge of its own as it is so thoroughly worsening all the above challenges. This global pandemic has worsened the slow progress to ‘Zero Hunger’ in Africa. Furthermore, the pandemic has a considerable negative effect on the economic development in Africa. Indeed, lately several households in Africa, with low levels of educational attainment and high dependence on labor income, experience an enormous real income shock that has visibly jeopardized their food security (54). Informal food traders are an essential part of a wider food system going from input suppliers to farmers to the final eaters (55). By far, the ‘informal food sector’ in Africa is still the highest employer to the young African population and has been greatly disorganised by COVID-19 pandemic. This sector is made up of small-scale owner-operated enterprises (e.g. selling food of various kinds, including street traders, hawkers, street restaurants, etc.) that employs more people than the formal food and grocery sector or even other sectors (55).

## Summary and future directions

Despite challenges, many African countries have made significant improvement in reducing stunting, wasting, and underweight among children < 5 years (56). African countries are striving towards ‘Zero Hunger’ by developing policies and involving several stakeholders. Notably, increased effort from the African nations, the African Union, and the international community is necessary to unlock the potential for ‘Zero Hunger’ on a long-term basis. This includes international partnership and collaboration on food systems, trade, health services, and climate change. Collaboration between African countries and several UN agencies and other development partners, as well as the African Union’s efforts to achieve ‘Zero Hunger’ in Africa, is important, but at risk of being insufficient. With only 9 years left to the 2030 SDGs target, all African countries should increase progress actions toward ‘Zero Hunger’ as well as champion the SDGs on the global stage, including the SDGs targets related to planetary health (climate, water, and land) where global efforts are crucial and to which the poorest people in the world are most vulnerable. African nations should continue and strengthen the research and development in sustainable and climate-resilient food and agricultural practices. Also, Africa must support the SDG agenda at the national and continental level to harness the synergies between the targets, including good governance, good health and well-being, gender equality, decent work and economic growth, as well as reduced inequalities.

## Conflict of interest and funding

The authors declare no potential conflicts of interest. PA and POI received funding from the Throne Holst Foundation and the Centre for Global Health at the University of Oslo’s Research ExceLlence and Innovation in Global HealTh (RELIGHT) program.
